# Differential Effect of Phosphorylation-Defective Survivin on Radiation Response in Estrogen Receptor-Positive and -Negative Breast Cancer

**DOI:** 10.1371/journal.pone.0120719

**Published:** 2015-03-12

**Authors:** Bisrat G. Debeb, Daniel L. Smith, Li Li, Richard Larson, Wei Xu, Wendy A. Woodward

**Affiliations:** Department of Radiation Oncology, University of Texas M.D. Anderson Cancer Center, Houston, TX, United States of America

## Abstract

Survivin is a key member of the inhibitor of apoptosis protein family, and is considered a promising therapeutic target due to its universal overexpression in cancers. Survivin is implicated in cellular radiation response through its role in apoptosis, cell division, and DNA damage response. In the present study, analysis of publically available data sets showed that survivin gene expression increased with breast cancer stage (*p* < 0.00001) and was significantly higher in estrogen receptor-negative cancers as compared to estrogen receptor-positive cancers (*p* = 9e-46). However, survivin was prognostic in estrogen receptor-positive tumors (*p* = 0.03) but not in estrogen receptor-negative tumors (*p* = 0.28). We assessed the effect of a survivin dominant-negative mutant on colony-formation (2D) and mammosphere-formation (3D) efficiency, and radiation response in the estrogen receptor-positive MCF7 and estrogen receptor-negative SUM149 breast cancer cell lines. The colony-formation efficiency was significantly lower in the dominant-negative survivin-transduced cells versus control MCF7 cells (0.42 vs. 0.58, *p* < 0.01), but it was significantly higher in dominant-negative population versus control-transduced SUM149 cells (0.29 vs. 0.20, *p* < 0.01). A similar, non-significant, trend in mammosphere-formation efficiency was observed. We compared the radiosensitivity of cells stably expressing dominant-negative survivin with their controls in both cell lines under 2D and 3D culture conditions following exposure to increasing doses of radiation. We found that the dominant-negative populations were radioprotective in MCF7 cells but radiosensitive in SUM149 cells compared to the control-transduced population; further, Taxol was synergistic with the survivin mutant in SUM149 but not MCF7. Our data suggests that survivin modulation influences radiation response differently in estrogen receptor-positive and estrogen receptor-negative breast cancer subtypes, warranting further investigation.

## Introduction

Survivin is the smallest member of the inhibitor of apoptosis protein (IAP) family at 16.5 kDa and is encoded by *BIRC5* (baculoviral inhibitor of apoptosis repeat-containing protein-5) [1]. It is implicated in the regulation of several cellular networks, and is prominent for its universal overexpression in human cancers. Survivin harbors many phosphorylation sites and interacts with a variety of different proteins, enabling its diverse functions that include its involvement in cellular division, apoptosis, intracellular signaling, and adaptation to unfavorable environments [2].

Survivin is clinically relevant in breast cancer and may be predictive of response to therapy. One of the seminal studies found that survivin is expressed in approximately 70% of breast carcinomas compared to no expression in adjacent normal tissue, and that survivin expression is a significant prognostic parameter of worse outcome in breast cancer patients [3]. Further, Kennedy and colleagues [4] found that nuclear survivin expression is prognostic of favorable outcome for breast cancer patients. A more recent study reported that survivin expression could function as a predictive biomarker of complete pathologic response to neoadjuvant chemotherapy in patients with stage II or stage III breast cancer [5]. Interestingly, survivin is a component of the 21-gene recurrence score validated in ER+ node-negative patients as a prognostic and predictive marker of recurrence and response to chemotherapy [6]. More recently, retrospective analysis of clinical trials from which these studies were validated revealed the recurrence score also predicts for risk of local recurrence among patients treated with lumpectomy and radiation [7].

Survivin, due in part to its role in apoptosis, and cell division, has long been proposed as a predictive factor for response to radiation therapy treatments, and anti-survivin treatments have been explored as possible radiosensitizers in preclinical studies [8]. More recently, it has been reported that survivin also plays a role in DNA double-strand break repair [9], adding another mechanism by which survivin may increase cellular radio-resistance. Several preclinical studies have shown that survivin is associated with radiation resistance in pancreatic cancer [10], colorectal cancer [11], melanoma [12], lung cancer [13], glioblastoma [14], and epidermoid carcinoma [15]. Further, several of these and other preclinical studies [16] [17] [18] [19] tested the efficacy of anti-survivin treatments—including the use of antisense oligonucleotides, siRNA, ribozymes, dominant-negative mutants, and small-molecule inhibitors—in combination with radiation. In each case, the combination treatment was more effective than radiation alone, and increased apoptosis as well as decreased cell survival and growth were observed in the combined regimen. In murine mammary epithelial cells, Woodward and colleagues [20] [21] reported that survivin is selectively upregulated following irradiation in stem cell-enriched populations; however, no group has specifically examined if survivin is a radiation resistance factor in breast cancer cells.

In the present study, we examined public gene expression datasets and report that although survivin expression is higher in estrogen receptor-negative (ER−) than estrogen-receptor-positive (ER+) breast cancer, it is only prognostic in ER+ breast cancer. Based on the differential impact that survivin expression has on overall survival in ER+ and ER− breast cancer patients, we hypothesized that survivin perturbation would exert different effects on an ER+ versus an ER− cell line. We evaluated how a phosphorylation-defective mutant of survivin (survivin-DN) affects apoptosis, self-renewal capacity, and radiation response in ER+ and ER− breast cancer cell lines.

## Materials and Methods

### Data Mining

*BIRC5* expression in different breast cancer patient cohorts was extracted from two public databases, Oncomine [22] and Gene Expression-Based Outcome for Breast Cancer Online (GOBO) [23]. In Oncomine, data was specifically extracted from The Cancer Genome Atlas, Bittner (unpublished), and Curtis [24] breast datasets. *BIRC5* expression was then stratified based on three characteristics: presence of invasive carcinoma, stage, and receptor status.

Breast cancer patient survival information (all breast cancer patients, ER+ patients, or ER− patients) was evaluated in the Kaplan-Meier Plotter (K-M Plot) [25] and GOBO public databases, where patients were stratified into groups of high and low *BIRC5* expression using a database-selected “cutoff” point.

### Cell culture

The estrogen receptor-positive breast cancer cell line MCF7 was acquired from ATCC and cultured in Modified Eagle Medium (MEM) supplemented with 10% fetal bovine serum, 0.1 mM nonessential amino acids, 1 mM sodium pyruvate, 1 μg/mL hydrocortisone, 5 μg/mL insulin, and 1% antibiotic-antimycotic. The triple-negative, inflammatory breast cancer cell line SUM149 was obtained from Asterand (Detroit, MI, USA) and was cultured in Ham’s F-12 media supplemented with 10% fetal bovine serum (FBS), 1 μg/mL hydrocortisone, 5 μg/mL insulin, and 1% antibiotic-antimycotic. Cell lines were maintained at 37°C in a humidified atmosphere (5% carbon dioxide).

### Construct

In order to generate a survivin dominant-negative mutant, an adenoviral construct with a survivin-T34A point mutation, kindly provided by Dr. Altieri and colleagues [26], was acquired. The green fluorescent protein from the pFUGW backbone [27] was removed, after which the survivin dominant-negative construct (survivin-DN) was cloned into the HindIII-BamHI site. pFUGW was used as a negative control throughout the study.

### Western blot

Western blots were run to validate induction of the survivin-DN construct and to evaluate caspase cleavage as a marker for apoptosis in both MCF7 and SUM149 cell lines. The lysate was collected with 1X RIPA lysis buffer (diluted from 10X RIPA from Cell Signaling, Danvers, MA) containing 1 uM phenylmethyl sulfonyl fluoride and was transferred to microcentrifuge tubes. The samples were rotated for one hour at 4°C and centrifuged at 10,000 *g* for 10 minutes. Fifty-μg aliquots of the protein lysate supernatants were electrophoresed on 4–20% gradient sodium dodecyl sulfate-polyacyrlamide gels (Invitrogen, Life Technologies, Carlsbad, CA) and transferred to polyvinylidene fluoride membranes (Bio-Rad Laboratories, Hercules, CA). The membranes were incubated in 5% non-fat milk for one hour at room temperature and then incubated at 4°C for 16 hours with the primary antibody: rabbit anti-survivin mAb (Cell Signaling #2808, Danvers, MA), rabbit anti-caspase 3 mAb (Cell Signaling #9662) and rabbit anti-cleaved capsase 3 mAb (Cell Signaling #9664). The membranes were then washed three times and incubated with the corresponding secondary antibody conjugated with horseradish peroxidase (Santa Cruz, CA) in 5% non-fat milk at room temperature. Next, the membranes were washed three times and immunoreactivity was detected by enhanced chemiluminescence. For all western blots, mouse anti-β-actin mAb (Sigma-Aldrich #A5316, St. Louis, MO) was used as a loading control.

### Cell cycle assay

Cell cycle analyses were performed as described previously [28]. Briefly, cells were enzymatically dissociated and centrifuged for 5 minutes at 4°C. After washing once with PBS, cells were fixed with 70% cold ethanol and were left overnight at 4°C. Cells were then centrifuged and resuspended in a Propidium Iodide solution (50 μg/mL Propidium Iodide). RNase (20 μg/mL) was added and samples incubated at 37°C for 1 hour. Samples were then immediately analyzed for DNA content using FACSAria II flow cytometer from Becton-Dickinson (BD Biosciences, San Jose, CA), and the distribution of cells across cell phases was analyzed using FlowJo software (Treestar, Ashland, OR).

### Mammosphere cultures

Cancer stem/progenitor cells can be enriched by propagating cells in serum-free, growth factor-enriched conditions—called mammospheres (3D) cultures in the case of breast cancer [29]. To generate mammospheres from MCF7 and SUM149 cells, 2 x 10^4^ cells/mL were cultured in serum-free MEM supplemented with 20 ng/mL basic fibroblast growth factor (Invitrogen), 20 ng/mL epidermal growth factor (Invitrogen), and B27 (Invitrogen) in six-well, ultra-low attachment plates.

### Clonogenic assays

The radiosensitivity of the survivin-DN construct in both monolayer (2D) and mammosphere (3D) cultures was evaluated as described previously [30]. For both 2D and 3D radiosensitivity assays, single cells from dissociated MCF7 and SUM149 monolayer cultures were seeded into 6-well tissue culture plates. The 6-well plates containing seeded cells were irradiated with γ-irradiation (0, 2, 4, or 6 Gy) four hours after plating with a ^137^Cs source (Shepherd Irradiator, J.L. Shepherd and Associates, San Fernando, CA). For 2D monolayer culture, the plates were incubated for 14 days, after which the colonies were stained with crystal violet and then counted manually. For 3D mammosphere cultures, the cells were incubated in mammosphere media for 7 days, and then the spheres were stained with MTT to improve visualization.

In a separate set of experiments, mammosphere cultures were incubated with either 10 nM Taxol (Cayman Chemical, Ann Arbor, MI) or 1 μM GSI (CalBiochem, Darmstadt, Germany). Spheres with a minimal size of 50 μm were counted using a GelCount colony counter (Oxford Optronix, Oxford, UK). Linear-quadratic survival curves were generated using SigmaPlot, version 8.0 (Systat Software, San Jose, CA).

### Tissue Staining

Primary MCF7 and SUM149 tumor xenograft tissue was used for immunohistochemical staining to detect survivin and phospho (pT34)-survivin with the rabbit anti-survivin mAb (Cell Signaling #2808, Danvers, MA) and rabbit anti-pT34-survivin (Santa Cruz, sc-23758) antibodies, respectively.

### Statistical Analysis

Statistical analyses were performed in GraphPad Prism version 6. Two-tailed Student’s *t* test was used to evaluate colony- and mammosphere-formation efficiency and to compare group means in the clonogenic assay, with *p* < 0.05 considered statistically significant.

## Results

**Fig 1:**
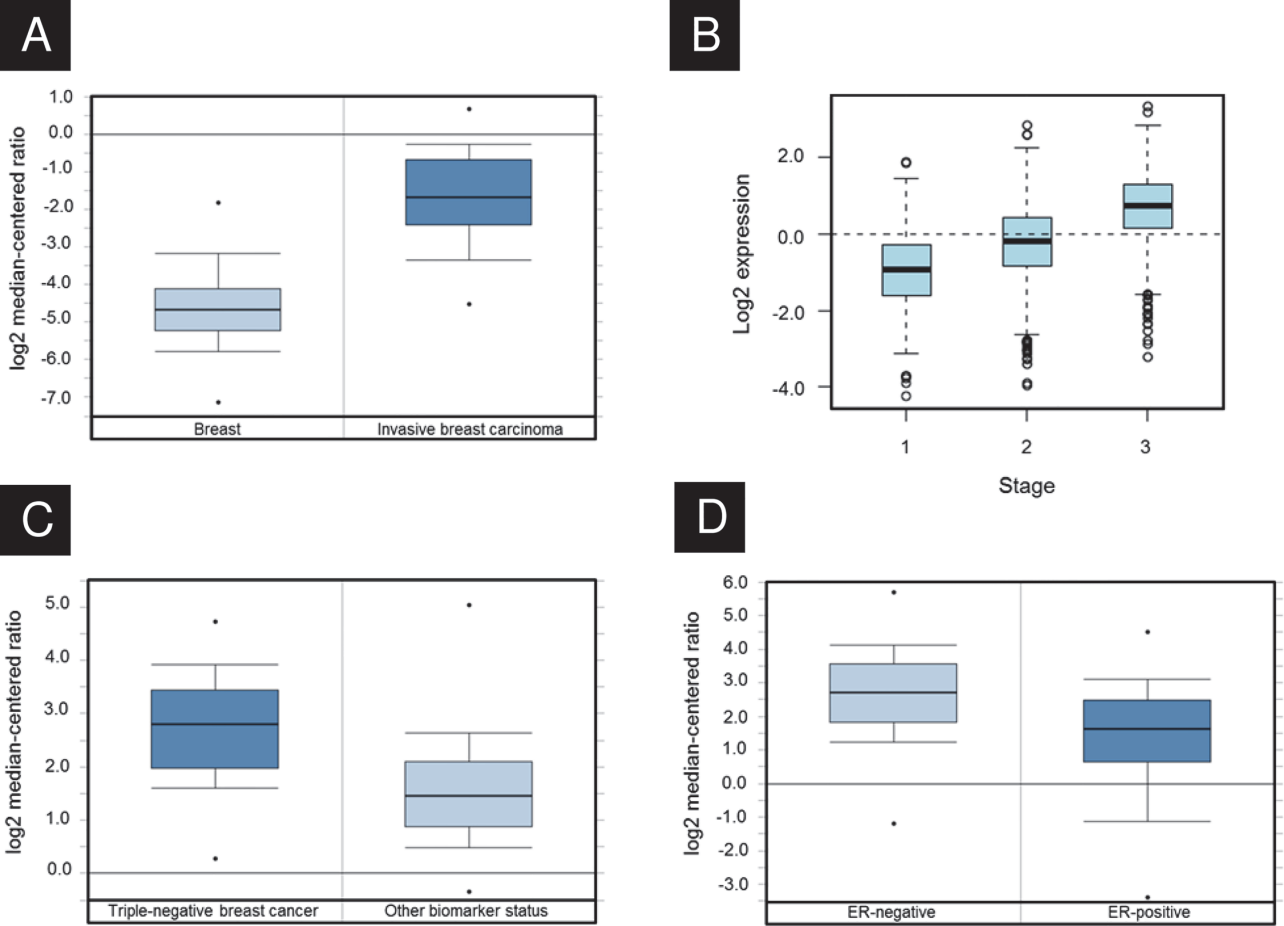
Survivin expression in breast cancer from public databases. **A)** Survivin expression is approximately seven-fold higher in invasive breast carcinoma compared to normal breast (p = 5.5e-31) from the TCGA data set in the Oncomine public database. **B)** From the GOBO public dataset, survivin expression increases with breast cancer stage (p < 0.00001). **C)** Survivin is expressed 2.3-fold higher in triple-negative breast cancer compared to all other molecular subtypes (p = 3.5e-8) in the Bittner breast data set in Oncomine. **D)** Similarly, survivin expression is 2.3-fold higher in estrogen receptor-negative compared to estrogen receptor-positive breast cancers (p = 9e-46) in the Curtis breast data set [24] in Oncomine.

We investigated the relevance of survivin to breast cancer by extracting *BIRC5* expression information from three public databases: Oncomine [22], Gene Expression-Based Outcome for Breast Cancer Online (GOBO) [23], and Kaplan-Meier Plotter (K-M Plot) [25]. We found that *BIRC5* was expressed significantly higher in invasive breast carcinoma compared to normal breast tissues (Fig. 1A, *p* = 5.5e-31) and increased with breast cancer stage (Fig. 1B, *p* < 0.00001). Moreover, *BIRC5* was expressed significantly higher in triple-negative breast cancer, a type of breast cancer known to be more aggressive and with poor prognosis, compared to all other combined subtypes (Fig. 1C, *p* = 3.5e-8). Furthermore, *BIRC5* was expressed over two-fold higher in estrogen receptor-negative (ER−) breast cancers compared to estrogen receptor-positive (ER+) breast cancers (Fig. 1D, *p* = 9e-46).

**Fig 2:**
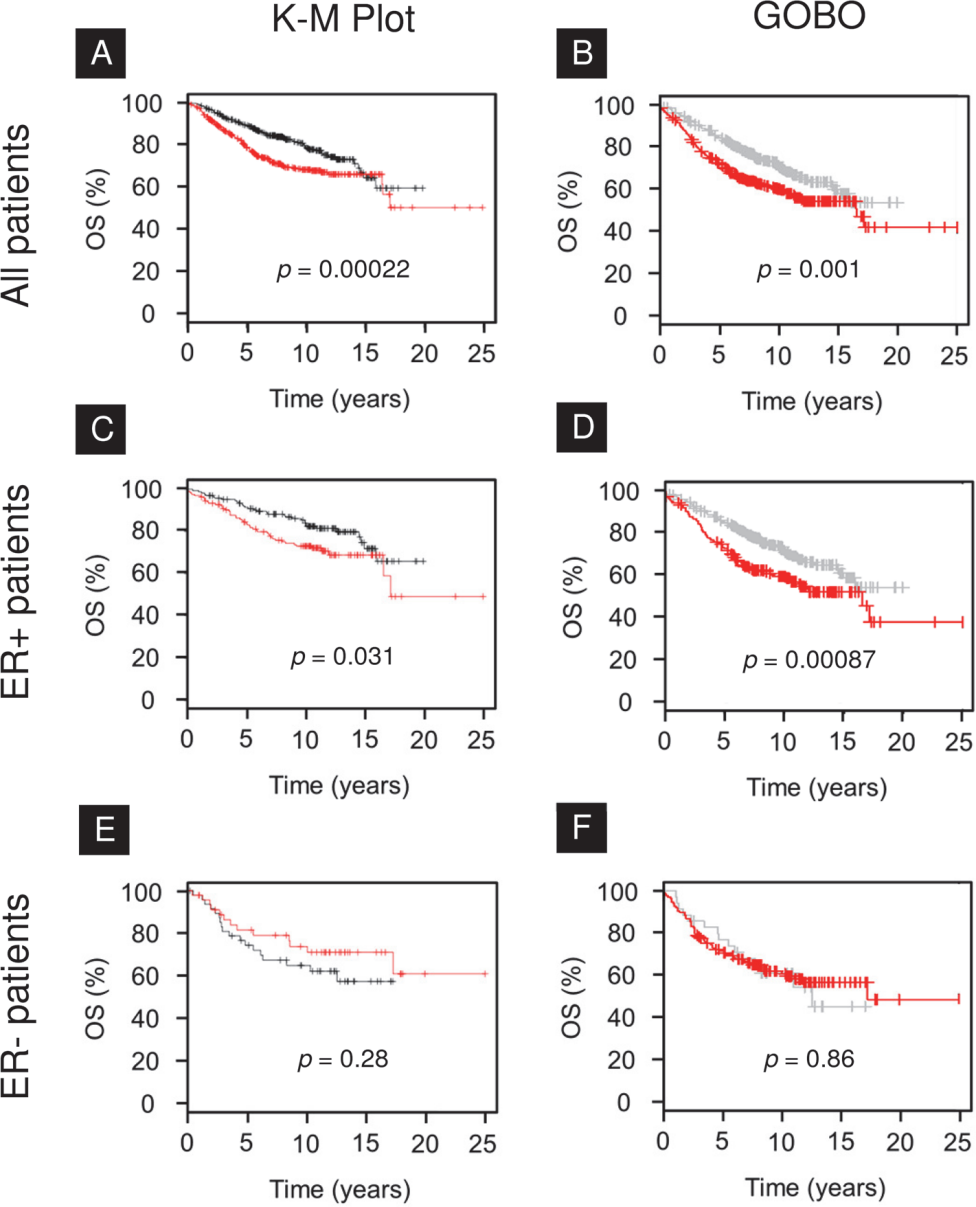
Overall survival in breast cancer stratified by survivin expression using two public databases. Kaplan-Meier Plotter (A,C,E) and Gene Expression-Based Outcome for Breast Cancer Online (B,D,F) data are shown. Red = high survivin expression at selected cutoff expression. **A,B)** High survivin expression is prognostic for poor outcome in all breast cancer patients. **C,D)** Likewise, high survivin expression predicts for poor outcome in patients with estrogen receptor-positive breast cancer. **E,F)** In patients with estrogen receptor-negative breast cancer, survivin expression is not associated with clinical outcome.

To determine whether survivin expression correlates with prognosis in patients with ER+ and ER− tumors, we analyzed two public breast cancer databases which had outcome data [22] [25]. In K-M Plot, we found that high *BIRC5* expression is associated with poor overall survival in all breast cancers patients (Fig. 2A, *p* = 0.0002) and in patients with ER+ breast cancer (Fig. 2C, *p* = 0.03), but was not associated with response in ER− patients (Fig. 2E, *p* = 0.28). Similar results were observed in the second database with respect to overall survival in all breast cancer patients, ER+ patients, and ER− patients (Fig. 2B, D, F).

**Fig 3:**
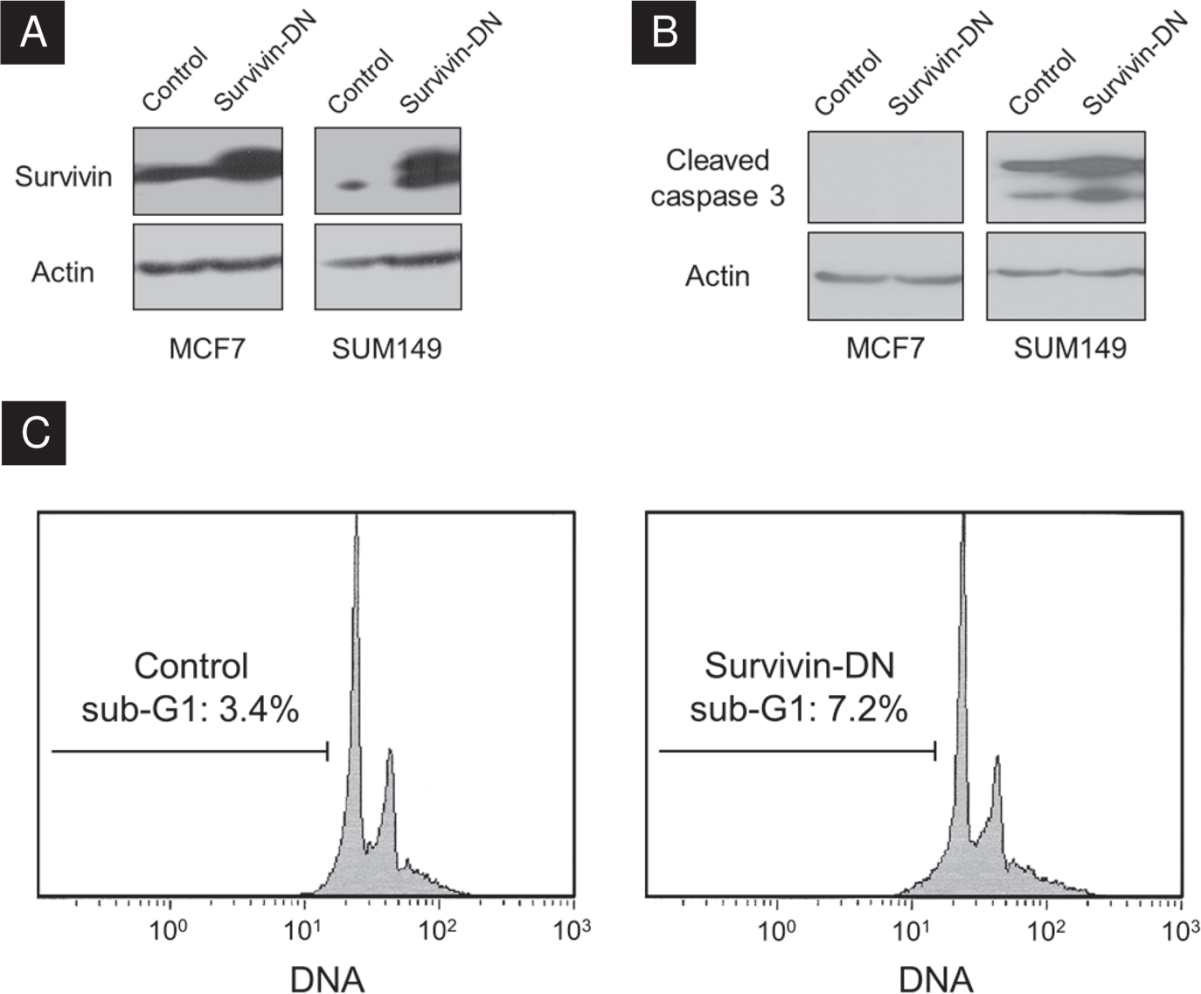
Cells transduced with the survivin dominant-negative construct display higher levels of apoptotic markers. **A)** MCF7 and SUM149 breast cancer cell lines were successfully transduced with the survivin dominant-negative construct, as shown by Western blot. **B)** Survivin-DN cells display greater levels of cleaved caspase 3 compared to the control; MCF7 shows no expression of caspase 3, consistent with the literature [32]. **C)** Survivin-DN cells have a greater fraction of sub-G1 (i.e. apoptotic) cells compared to the control, when stained with Propidium iodide.

To functionally assess whether survivin plays a significant role in the radiation response of breast cancer, we generated a survivin dominant-negative construct by cloning a T34A mutant into a pFUGW lentiviral backbone. As has been observed in T34A-transfected 293T cells by Altieri’s group [26], we found induction of the dominant-negative mutant in MCF7 and SUM149 cell lines (Fig. 3A). Normally, this threonine residue would be phosphorylated by p34(cdc2)-cyclin B1, which is important in survivin protein stability and trafficking [31]. We first assessed the effect of the dominant-negative mutant on the frequency of apoptosis in MCF7 and SUM149 cell lines with caspase cleavage and cell cycle assays. In the caspase cleavage assay, the survivin-DN-transduced SUM149 cells showed greater levels of caspase cleavage compared to the pFUGW control (Fig. 3B). MCF7 did not express caspase 3, consistent with the literature [32].

In cell cycle assays we stained both cell lines with Propidium Iodide, and measured the number of cells in the sub-G1 phase of the cell cycle as a surrogate for apoptosis. In the SUM149 survivin-DN population, 7.2% of cells were in the sub-G1 phase, compared to only 3.4% of cells in the control population (Fig. 3C). Similarly, there was also a higher percentage of cells in sub-G1 phase for the survivin-DN population compared to the control in MCF7 cells (Fig. 3D).

**Fig 4:**
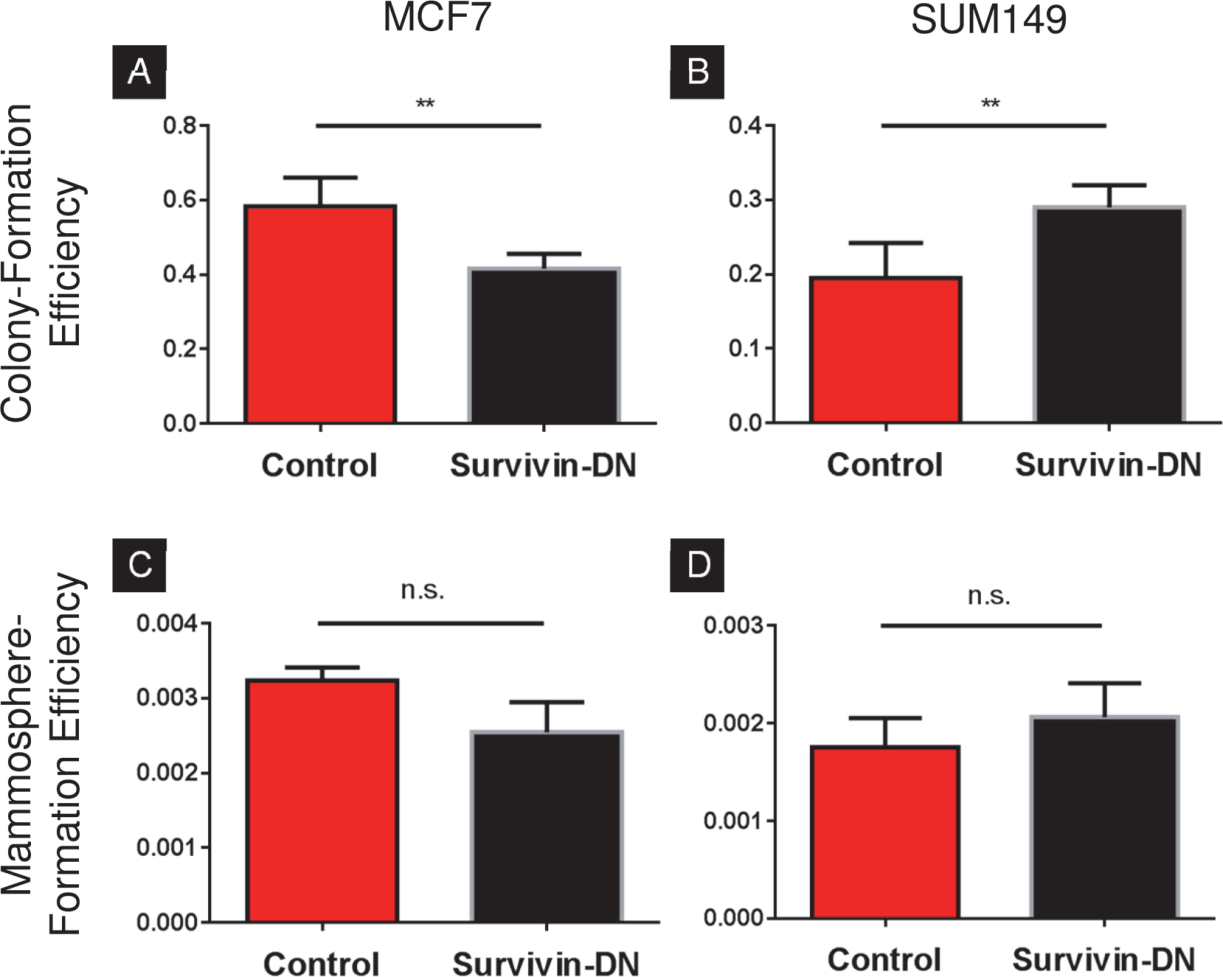
Colony- and mammosphere-formation efficiency in MCF7 and SUM149 breast cancer cell lines. **A)** In MCF7, the FUGW control forms significantly more colonies than the survivin-DN-transfected cells (p < 0.01). **B)** In SUM149, the survivin-DN cells form significantly more colonies than the control (p < 0.01). **C,D)** In both MCF7 and SUM149, there is no statistical difference in mammosphere-formation efficiency between the control and survivin-DN clone. Error bars indicate standard deviation.

We then evaluated the effect of the T34A survivin mutation on colony- and mammosphere-formation assays. In the ER+ MCF7 cell line, colony-formation efficiency was significantly lower in the survivin-DN population as compared to the control (Fig. 4A, 0.42 vs. 0.58, *p* < 0.01). In the ER− SUM149 cell line, however, the survivin-DN cells had significantly greater colony-formation efficiency (Fig. 4B, 0.29 vs. 0.20, *p* < 0.01). No significant difference in mammosphere-formation efficiency was observed between survivin-DN and control in either cell line (Fig. 4C, 4D, *p* > 0.05).

**Fig 5:**
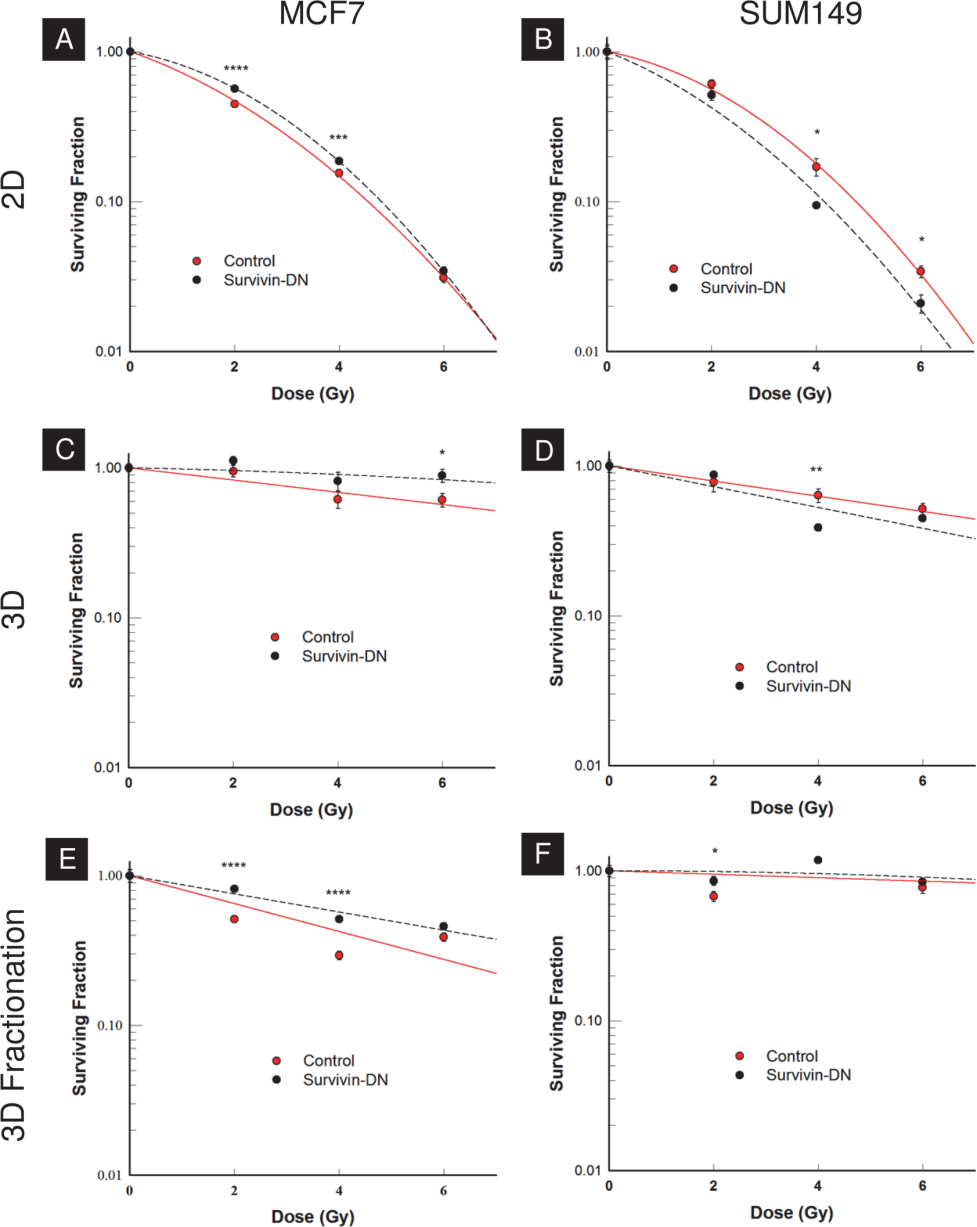
Representative figures for monolayer and mammosphere clonogenic assays in MCF7 and SUM149. **A,C,E)** MCF7 survivin-DN cells are radio-protective in monolayer cultures, mammosphere cultures, and also mammosphere cultures undergoing a fractionated regimen. **B)** SUM149 survivin-DN cells are radiosensitive compared to the control in monolayer cultures. **D,F)** SUM149 survivin-DN cells show no statistical difference in response to radiation, for both mammosphere cultures and mammosphere cultures exposed to a fractionated regiment. Error bars indicate standard deviation.

Next, we sought to investigate how the survivin-DN construct affects radiation sensitivity in MCF7 and SUM149. For both cell lines, clonogenic assays were performed with single-dose irradiation in monolayer and mammosphere conditions, and with fractionated radiation under mammosphere conditions. In all three conditions in MCF7, survivin-DN was radio-protective, with the survivin-DN-transduced MCF7 cells showing more resistance to irradiation than control-transduced MCF7 cells (Fig. 5A, C, E). In the SUM149 monolayer cultures, however, survivin-DN-transduced cells were radiosensitized compared to the control-transduced cells (Fig. 5B, 5D). Radiation response between different groups can also be compared by calculating surviving fraction at 2 Gy (SF2) values. For MCF7, survivin-DN was slightly radioprotective under all plating conditions, while survivin-DN was slightly radiosensitized compared to the control in SUM149 (Table 1).

**Table 1:** Survival Fraction at 2 Gy for survivin-DN in MCF7 and SUM149.

	**Control**	**Survivin-DN**
**MCF7 2D**	0.23 (*13*)	0.30 (*16*)
**MCF7 3D**	0.60 (*23*)	0.63 (*19*)
**SUM149 2D**	0.17 (*08*)	0.15 (*12*)
**SUM149 3D**	0.89 (*19*)	0.82 (*20*)

**Fig 6:**
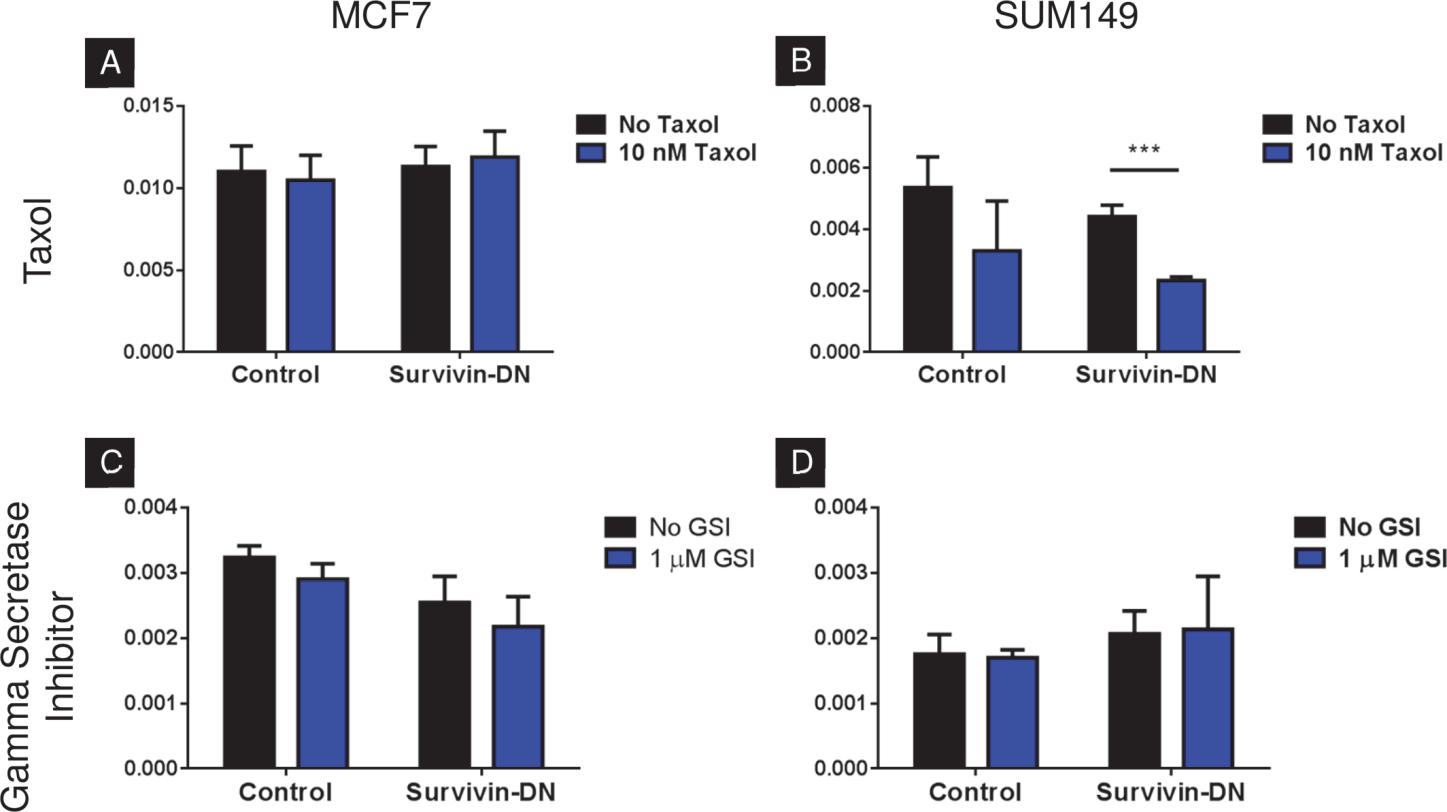
Mammosphere-formation efficiency in MCF7 and SUM149 when selected drugs are administered to survivin-DN cells. **A,C)** Neither Taxol nor gamma secretase inhibitor decrease mammosphere-formation efficiency in MCF7 control or survivin-DN cells. **B)** SUM149 survivin-DN cells are sensitized by treatment with 10 nM Taxol (p < 0.001). **D)** Gamma secretase inhibitor shows no effect on mammosphere formation in SUM149 control or survivin-DN cells. Error bars indicate standard deviation.

We investigated the synergy of survivin perturbation and chemotherapy by adding Taxol to both the control and survivin-DN populations in MCF7 and SUM149 cell lines. In Taxol-treated cells, there were no significant differences between control and survivin-DN MCF7 cells (Fig. 6A, *p* > 0.05). In SUM149 cells, however, the combined regimen with Taxol and survivin-DN significantly decreased mammosphere-formation efficiency compared with survivin-DN alone (Fig. 6B, 0.002 vs. 0.004, *p* < 0.001). We also evaluated the synergy of a gamma secretase inhibitor with survivin-DN in both cell lines, but no significant differences were observed (Fig. 6C, 6D, *p* > 0.05).

After staining SUM149 and MCF7 tissues for pT34-survivin in order to quantify the background activity of survivin, no differences were observed (S1 Fig.). Further, the total survivin and phospho-survivin was localized in the nucleus in both the control and survivin-DN SUM149 clones (S2 Fig.).

## Discussion

Here we report that T34A, phosphorylation-defective survivin reduces colony and mammosphere formation in the ER+ MCF7 cell line but not in the ER− SUM149 cell line. Conversely, the phosphorylation-defective survivin synergizes with taxol and radiation in an ER− cell line, SUM149, but not in the ER+ MCF7, suggesting that the primary function of survivin in these cell lines and potentially in ER+ and ER− tumors is different.

We extracted information from three public databases regarding expression of *BICR5*, the gene that encodes survivin. We found that increased *BIRC5* expression is associated with advanced stage and ER− disease, but is prognostic only in ER+ breast cancer patients. These data raise important questions about targeting survivin in ER+ and ER− tumors and lead to the speculation that the dominant function of survivin in ER+ tumors may be promotion of progression, while in ER− tumors it may be regulation of response. This is consistent with the well-documented clinical paradox of greater response but worse overall outcomes in patients with ER− breast cancer who receive neoadjuvant chemotherapy.

Evasion from apoptotic death is a key mechanism in the response to therapy, in effect resistance of cancer cells to ionizing radiation and chemotherapy. Indeed, it is considered a hallmark of cancer. Changes in the activity of apoptotic pathways thus influence the response to anti-cancer treatments, and disruption of these pathways by interfering with anti-apoptotic factors is an attractive strategy to counteract therapeutic resistance. Survivin, an inhibitor of apoptosis protein that is universally overexpressed in human cancers, represents one such target.

To assess the relevance of survivin function to colony formation and radiation response in breast cancer, we employed the T34A phosphorylation-defective survivin in ER+ MCF7 and ER− SUM149 breast cancer cell lines, performing apoptosis, colony- and mammosphere-formation, and clonogenic assays. As we have reported before [30], the colony-formation and clonogenic assays performed under monolayer conditions may not fully reflect the effect of treatment on the stem and progenitor cell fraction, which is often enriched after treatment. The mammosphere assay, however, is thought to select for the self-renewing, stem cell fraction, and because of the greater relative resistance of stem cells compared to more differentiated cells to conventional treatments, we observed greater sensitivity of the monolayer cultures to irradiation compared to the mammosphere cultures as we and others have published previously [33,34,35]. Interestingly, there were greater differences in radiosensitivity between the control and phosphorylation-defective survivin populations in monolayer cultures as compared to mammosphere cultures; however, this may be due to the extreme radioresistance of the 3D cultured cells. Finally, we explored the combination of the survivin dominant-negative mutant with either Taxol or a gamma secretase inhibitor, as Notch signaling has been reported to increase survivin levels in basal-like breast cancer but not in ER+ breast cancer [36,37]. Absolute differences were detected only in the ER+ cells; however, these were modest and not significant.

Numerous attempts to target survivin in preclinical breast cancer models have been successful. One group employed the T34A phosphorylation-defective survivin to mitigate the growth and metastatic potential of a 4T1 mouse model of breast cancer [38]. In a separate study, the administration of a small-molecule survivin suppressant led to a regression of the primary and reduced spontaneous metastases in the triple-negative mouse model of breast cancer [39]. The results from these studies do not parallel our findings in the databases, in which survivin was not prognostic in estrogen receptor-negative breast cancer; further, we found that the phosphorylation-defective survivin increased colony-formation efficiency in the triple-negative SUM149. Nevertheless, all results consistently highlight the potential clinical benefit to abrogating survivin function in breast cancers.

A relationship between survivin and estrogen has been reported previously. Frasor et al. [40] observed that estradiol upregulates survivin expression in the ER+ MCF7. A mechanism was established by Sayeed and colleagues [41], who found in chromatin immunoprecipitation assays that estrogen upregulates survivin through a p53-dependent mechanism. ERα interacts with p53 bound to the promoter of survivin, inhibiting p53-mediated transcriptional repression of survivin and opposing p53-mediated apoptosis in breast cancer cells. Span et al. [42] suggested that higher survivin expression in ER− cells may be due to a difference in the cellular origin of ER− (as compared to ER+) tumors rather than due to differences in estrogen-mediated survivin expression. Chen et al. [43] observed that, among twenty endometrial hyperplasia patients that responded to progestin therapy, there was a twenty-fold decrease of nuclear survivin expression and eight-fold decrease in cytoplasmic survivin expression; conversely, there was no change in survivin expression among non-responders. These data implied that high survivin in ER+ cells is a function of unopposed estrogen in ER+ tumors, and that treatment-responsive tumors reduce survivin expression.

In conclusion, we describe the disparate effect of a dominant-negative form of survivin on the colony-forming potential and radiation response in ER+ and ER− breast cancer cell lines. This study provides insight into the interaction between estrogen and survivin and highlights further study is warranted regarding survivin targeting to enhance therapy in ER− disease versus reduce progression in ER+ disease.

## Supporting Information

S1 Fig Survivin activity in MCF7 and SUM149 xenograft tissue. (TIF)

S2 Fig Staining of SUM149 primary tumor xenograft for survivin. (TIF)
