# Supplementary figures and images for: Differential Effect of Phosphorylation-Defective Survivin on Radiation Response in Estrogen Receptor-Positive and -Negative Breast Cancer
Source: PLoS One. 2015 Mar 12;10(3):e0120719. doi: 10.1371/journal.pone.0120719 (PMC4357387; doi:10.1371/journal.pone.0120719)

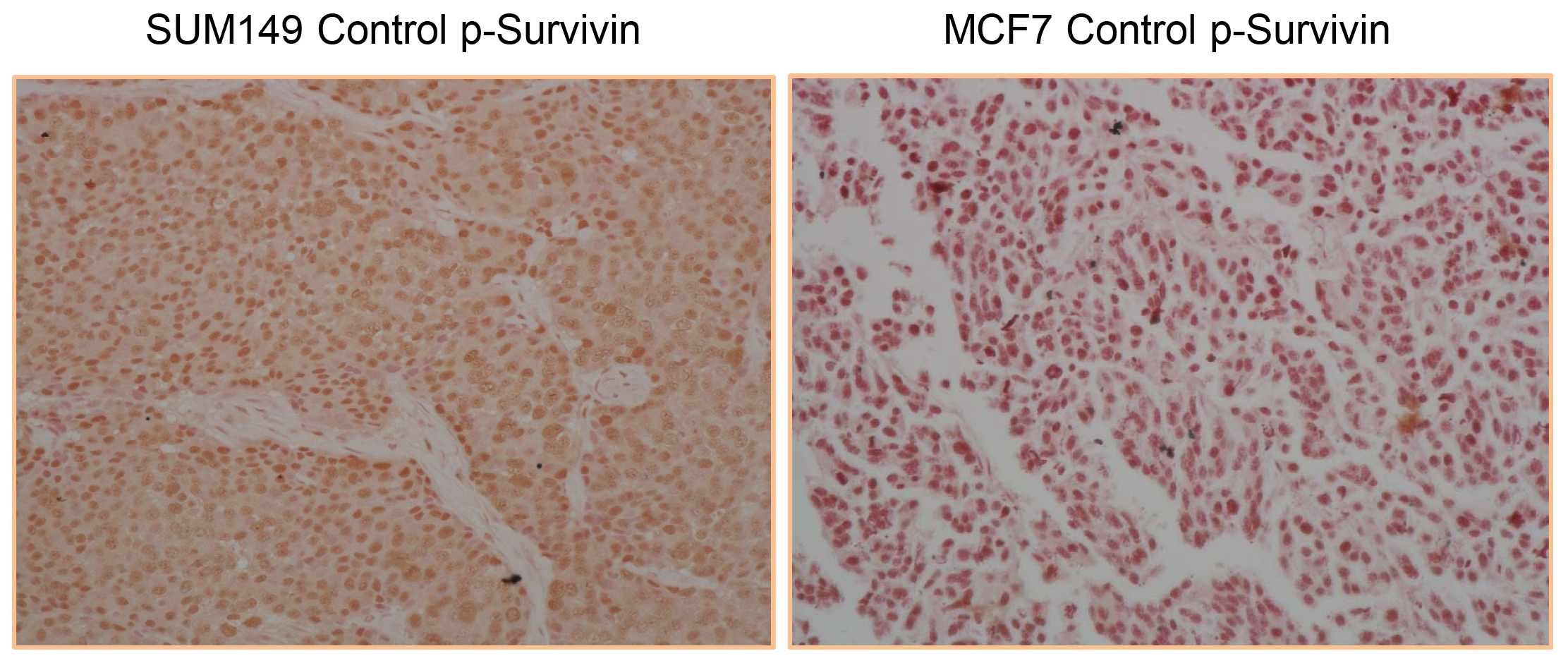

Supplement: S1 Fig — (TIF) [file pone.0120719.s001.tif]

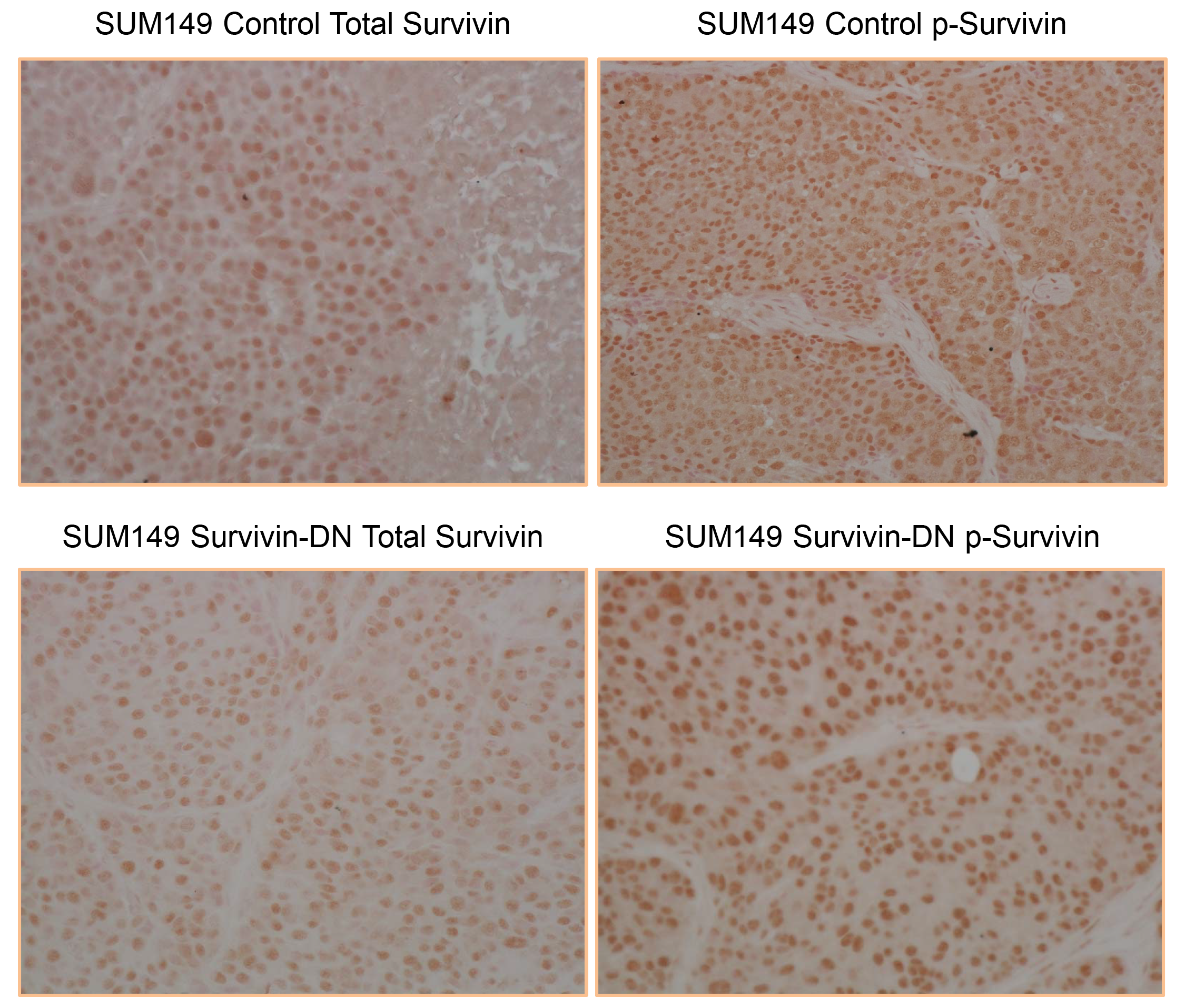

Supplement: S2 Fig — (TIF) [file pone.0120719.s002.tif]
